# Sulphuric Ether—Letheon

**Published:** 1847-04

**Authors:** 


### SELECTIONS

FROM

### AMERICAN AND FOREIGN JOURNALS.

---

*Sulphuric Ether—Letheon.*—Since the publication of Dr. Royle's work, this substance has acquired great celebrity in our country, under the name of *letheon*, for producing insensibility to pain during surgical operations. It was first employed for this purpose by Dr Morton, a dentist of Boston, at the suggestion of Dr. C. T. Jackson, of that city, in operations on the teeth. Patients are brought under its influence by inhaling the vapor from a very pure preparation of the ether. Drs. Warren and Hayward have operated on patients, to whom the ether was administered by Dr. Morton, by means of a peculiar apparatus, and there was little or no pain produced by the knife. The effects produced differed in different persons; in some there seemed to be complete insensibility; in others, considerable anxiety and agitation were produced, but they usually stated, after the termination of the operation, that they had felt no pain, but that their groans, &c. had been produced by unpleasant dreams, &c.; in others, the insensibility was only partial, but the pain was decidedly mitigated. There was no unpleasant effect in any of the cases reported by Dr. Warren.

The experiments in Baltimore were not so favorable, in three cases, in which it was permitted to be administered by Prof. Smith, it not only did not succeed, but acted injuriously. It is said to have failed in some cases in New-York. The cases, however, in which it has been completely successful are so numerous as to place its efficacy, in a majority of cases, beyond a doubt.—*South. Jour. Med. and Pharm.*

---

*Use of the Letheon in London.*—As intimated in the Journal heretofore, the ethereal inhalation seems destined, notwithstanding the jealousy of some and the doubts of many at home, to a general favor and use abroad. The following is some of the information on this point received by the last arrivals from England.—*Boston Med. Jour.*

*Middlesex Hospital.*—On Monday last, at the Middlesex Hospital, the efficacy of the ether was put to as severe a test as it has yet been subjected to. A man of 68 had been admitted with symptoms of stone and diseased bladder; so much pain, straining and struggling attended the attempts at sounding, that it was with difficulty satisfactorily accomplished. The vesical tenesmus was incessant, amounting to total incontinence of urine. Endeavors were made for several weeks to allay this extreme irritability, so that some urine might be retained or some water received as an injection, but in vain, neither could be endured; lithotrity was consequently out of the question; and Mr. Arnott determined to perform the operation of Lithotomy, unpromising as the case was, but, if possible, whilst the patient was under the influence of the ether. In seven minutes from the commencement, but in reality only two from the effectual inhalation, its influence was obtained. The catheter was then introduced, and some water attempted to be injected, but not above two or three ounces could be borne, and this, retained by pressure, was ejected immediately on the introduction of the staff, which, owing to the state of the parts, was effected with some difficulty and consequent delay; the bladder was cut into; the stone was grasped at once, but crumbled under the forceps, requiring their re-introduction several times; the scoop was employed to remove calculous matter like mortar; and, lastly, the bladder was injected four or five times, so as to wash it out. During the whole time, from first to last, the patient gave not the slightest indication of suffering; indeed, it was not until he was removed to bed and had been some time in it, and taken some brandy and ammonia, that he did so, and then of soreness merely. Nor was the influence of the ether limited to this, its anodyne effect was maintained during the evening, the man remaining in a dreamy and "very comfortable state," as he termed it. He declared that he suffered no pain; he knew that something was being done, but he recollects nothing distinctly "after blowing the horn." Up to this time, Wednesday evening, he is going on very favorably. Without the ether the pain in this case must have been most severe, and, from the circumstances mentioned, of more than ordinary duration, but happily the patient was spared it all. The apparatus employed was one invented by Mr. Bell, chemist, of Oxford street, who was present, and assisted Mr. Tomes in its application.—*Med. Gazette.*

*The Cæsarean Operation.*—The subject of this case, a dressmaker, æt. 27, of a mild disposition, is only four feet one inch in height, on account of great distortion of the pelvis and lower limbs from rickets during childhood. Her general health is good. On the evening of the 7th of April, 1846, while under temporary excitement, she had connection once with a young man lodging in the same house. She was not aware of being pregnant until the seventh month, when she consulted a surgeon, who, conscious of her dangerous position, sent her to Mr. Skey, under whose care she was admitted into St. Bartholomew's Hospital. An accurate examination was then made by several distinguished accoucheurs, who were unanimous in their opinion that embryotomy would be impracticable on account of the extreme narrowness of the antero-posterior diameter of the pelvis. It was, therefore, recommended no operative proceeding should be adopted until the full period of utero-gestation; and that the Cæsarean section would then be the most proper measure. The nature of the case being fairly and fully explained to the patient, she readily consented to undergo any operation which offered the best chance of relief. At 2 o'clock in the morning of the 25th of January, she was awakened from sleep by the commencement of labor. The membranes gave way soon afterwards, and the pains increased. Mr. Skey, with several accoucheurs, made an examination, *per vaginam*, at half past 4, A. M. The os uteri was at that time very little dilated. A second examination was made at half past 7 o'clock. The os uteri was still in the same condition, but the labor pains were rapidly increasing. The operation was, therefore, no longer delayed. The vapor of ether was inhaled by the patient for six minutes before its effects were manifest; an incision eight inches in length was made down to the linea alba, commencing two inches above the umbilicus, and terminating two inches and a half above the pubes. The linea alba was then divided to the same extent on a broad director. The uterus was fairly exposed, inclining to the left. Adequate pressure over the front and sides of the abdomen was necessary to prevent protrusion of the intestines. An incision from five to six inches in length, was then made into the long axis of the uterus, from which a well-formed, healthy-looking female child was easily removed. The placenta was extracted shortly afterwards. Thus far the operation occupied six minutes. Immediate contraction of the uterus to one half its previous size followed the removal of the child. The free venous hemorrhage which took place from its cut surface was arrested by cold water and pressure between the hands. In half an hour the uterus had contracted to such a size as to render its replacement within the abdomen safe. Accordingly, with the sanction of Drs. Rigby, Ferguson, Moore, P. Smith, and others, the incision in the abdomen was brought together by eleven sutures. Broad strips of plaster were applied to support the muscles, and cotton wool placed on the abdomen with a flannel roller over the whole. It may be as well to observe that the inhalation of the ether produced insensibility to the pain of the first incision. Its prolonged exhibition was not allowed, lest it might possibly interfere with the contraction of the uterus.—*Ibid.*

*Ether applied to Veterinary Science.*—The vapor of sulphuric ether has, we hear, been employed at the Royal Veterinary College, Camden-town, on a sheep and a horse, with the most decided success. The first-named animal was affected, and had been for many months with an incurable disease of the hock-joint. The pain was so severe that the poor sheep was quite unable to put her foot to the ground without experiencing much suffering. On being brought into the theatre she was caused to inhale the vapor of ether through a tube, and in about five minutes after it was evident she was under its influence. The leg was then amputated by Mr. Simonds at the thigh, without the slightest indication of any pain whatever. The operation occupied about six minutes, and within twenty minutes from the commencement the animal was removed from the theatre and restored to sensation and consciousness. The horse was laboing under a chronic affection of the near fore-foot, commonly known by the name of the "naricular disease," for which the operation of "unnerving" is generally resorted to as a remedy. This is necessarily a very painful operation, and oftentimes the operator has to contend against the violent struggles of the animal, particularly at the instant when the division of the nerve is effected. In this case the ether vapor was inhaled for about thirteen minutes, when the horse fell forwards, and the nerve on each side of the leg was divided by Mr. Spooner without the least manifestation of pain; a slight convulsive action of the limb, similar to that which takes place when a nerve of a recently killed animal is cut through, alone giving indication of any sensation. Within twenty-three minutes this animal also had perfectly recovered from the effects of the ether. No restraint whatever was resorted to to keep the animals in the required position for these operations, and the inhaler employed was not one invented for the purpose, but an apparatus temporarily adjusted by Mr. Morton until a more perfect one was obtained.

*Pharmaceutical Society, January 13th,* 1847.—The lecture-room of the Society was crowded this evening with members of the Society and medical men, to hear a paper on Mr. Squire's instrument for the inhalation of the vapor of ether, and to see and to hear descriptions of other instruments for the same purpose.

Mr. Squire observed, this is now the all-engrossing subject, and public attention is daily called to the several operations which have been performed without the accompaniment of severe pain, heretofore experienced: some failures are also recorded, without stating the particulars as to their cause. In constructing the apparatus, I have taken into the account the weight of the vapor of ether, and have placed the tube near the bottom part; considering, also, that its stimulating effects on the air-passages cause coughing, I have regulated the supply of air to dilute it at the commencement of inhalation, with means to increase its strength until the patient is able to bear its full force. To secure a full supply I have filled the upper vessel with sponge, and have also a collar of sponge around its descending tube at the bottom of the lower vessel, which imbibes any liquid ether in excess, and also serves further to impregnate the air in its passage to the tube for inhalation. The apparatus is supplied with ether at the top by a funnel, and falls in a shower on the sponge; a valve is placed in this part, to admit air, and closes to prevent the escape of ether. In the other parts, Reed's patent valves (for the improvement of which he has recently taken out a patent) are, by his permission, used in this apparatus, and I trust it may serve the very useful purpose that its original was so successful in.

After Mr. Squire had exhibited and explained his instrument, Mr. Hooper gave an account of his apparaturs, or rather, of improvements which he had made in the instrument, suggested by Dr. Boott and Mr. Robinson, and which had received their sanction. It would be unprofitable to follow the various remaining describers of instruments which were exhibited, many of which had not been tested in practice; they were really so numerous, that it would appear that the whole scientific portion of the members of the society, as well as that of many others, had been employed in inventing and contriving means for administering the vapor of ether. The modifications attempted were from the most elaborate and complicated pieces of machinery, to mere bladders with an elastic tube and stop-cock, the latter having the advantage of not being protected by either "caveats" or "patents."

The object of this exhibition, at least, was answered, for each had an opportunity of exhibiting "his adopted," and, like a fond parent, saw advantages in his own offspring which he failed to find in that of others.

We may fairly sum up the results of the evening's "exhibition," in observing, with the President, that each form of apparatus exhibited had appeared to answer the purpose for which it was intended; that the simplest apparatus, if effective, must eventually be the most useful, and that time and experience only could demonstrate, amongst the many competitors, which form of instrument was the most likely to be generally useful.—*London Lancet.*

